# Comprehensive Analysis of Prognostic Value and Immune Infiltration of Chromobox Family Members in Colorectal Cancer

**DOI:** 10.3389/fonc.2020.582667

**Published:** 2020-09-04

**Authors:** Qingshang Li, Yi Pan, Zhijun Cao, Shuliang Zhao

**Affiliations:** Division of Gastroenterology and Hepatology, Key Laboratory of Gastroenterology and Hepatology, Ministry of Health, School of Medicine, Shanghai Institute of Digestive Disease, Renji Hospital, Shanghai Jiao Tong University, Shanghai, China

**Keywords:** bioinformatics analysis, chromobox family, colorectal cancer, biomarker, prognosis

## Abstract

**Objective:** Colorectal cancer (CRC) is one of the common malignant tumors worldwide. Chromobox (CBX) family proteins are important components of epigenetic regulation complexes and are implicated in the development of multiple cancers by blocking differentiation and promoting proliferation. However, little is known about the function of distinct CBX proteins in colorectal cancer.

**Methods:** Oncomine, Gene Expression Profiling Interactive Analysis (GEPIA), Kaplan-Meier plotter, cBioPortal, GeneMANIA, and TIMER were utilized to analyze differential expression, prognostic value, genetic alteration and immune cell infiltration of CBXs in colorectal cancer patients.

**Results:** The expression levels of CBX1/2/3/4/5 and CBX8 were significantly elevated in CRC tissues, whereas the expression levels of CBX6/7 were reduced. CBX3 was significantly associated with the clinical cancer stage and short disease-free survival (DFS) in CRC patients. High mRNA expression of CBX5/6 was associated with short overall survival (OS) in rectal cancer patients. CBX3/5/6 could be potential prognostic biomarkers for the survival of CRC patients. Moreover, the functions of the differentially expressed CBXs were primarily related to the SUMOylation of DNA methylation proteins and chromatin organization and may regulate the pluripotency of stem cells. The expression of CBXs were significantly correlated with the infiltration of diverse immune cells, including six types of CD4+ T cells, macrophages, neutrophils, B cells, CD8+ T cells, and dendritic cells in colon cancers and rectal cancers.

**Conclusions:** Our study may provide novel insights for the selection of prognostic biomarkers of CBX family in colorectal cancer.

## Introduction

Chromobox (CBX) family proteins are canonical components of polycomb group (PcG) complexes, which transcriptionally repress target genes by modifying chromatin (1). The CBX proteins are all involved in various biological processes, such as maintenance of pluripotency and self-renewal in embryonic stem cells, cell fate decisions, and developmental programs controls. Recently, The CBX proteins are widely recognized and valued for their roles in occurrence and progression of diverse tumors.

Eight members of the CBX protein family have been identified, each of which has a conserved structure: a single N-terminal chromodomain (2). They can be divided into two subgroups based on their molecular structure: the HP1 group (including CBX1, CBX3, and CBX5) and the Pc group (including CBX2, CBX4, CBX6, CBX7, and CBX8) (2).

In previous reports, some of CBX genes and proteins have been characterized and studied via general expression profile and the misregulation of some CBX proteins associated with various cancer types, especially breast cancer, liver cancer, and gastric cancer. However, the functions and prognostic roles of distinct CBX family members in colorectal cancer (CRC) remain unknown and elusive.

Colorectal cancer is one of the most common cancers and remains one of the leading causes of cancer death worldwide (3, 4). Therefore, we extended the research field to CRC based on a variety of large databases, with the purpose of determining the potential oncogenic values of distinct CBX family members in CRC.

## Materials and Methods

### Oncomine

The mRNA levels of distinct CBX family members in diverse cancer types were determined through analysis in Oncomine (www.oncomine.org), a publicly accessible online database providing powerful, genome-wide expression analysis with cancer microarray information (5). In this study, a *p*-value < 0.01, a fold change of 2, and a gene rank in the top 10% were set as the significance thresholds. Student's *t*-test was used to analyze the difference in the expression of CBXs in CRC.

### Gene Expression Profiling Interactive Analysis (GEPIA)

GEPIA (http://gepia.cancer-pku.cn/index.html) is a newly developed analytical tool using a standard processing pipeline and consist of thousands of tumors and normal tissue samples data (6). In this study, differential gene expression analysis was performed to make a comparison of tumor and normal tissues, pathological stage analysis, and correlative prognostic analysis through GEPIA. Student's *t*-test was used to generate a *p*-value for the expression or pathological stage analysis. Additionally, patient survival analysis was also performed using a Kaplan-Meier curve for further verification.

### Kaplan-Meier Plotter

The prognostic value of the mRNA expression of distinct CBXs in rectal cancer patients was analyzed by using Kaplan-Meier plotter (http://kmplot.com/analysis/), in which information about the association of gene expression with the survival of patients with liver cancer, breast cancer, ovarian cancer, lung cancer, and gastric cancer could be easily accessed (7). Information regarding the number-at-risk cases, median values of mRNA expression levels, HRs, 95% CIs and *p*-values can be found at the K-M plotter webpage. A statistically significant difference was considered when the *p*-value was < 0.05.

### cBioPortal

cBioPortal (www.cbioportal.org) is a comprehensive web resource, providing us with visual and multidimensional cancer genomics data (8). Based on The Cancer Genome Atlas (TCGA) database, genetic alterations, and the network module of CBXs were obtained from cBioPortal.

### GeneMANIA

GeneMANIA (http://www.genemania.org) is a resource rich website containing gene information, analyze gene lists and prioritize genes for functional assays with a high accuracy of prediction algorithm (9). We used it to weight that indicate the predictive value of CBXs.

### String

STRING (https://string-db.org/) is a website about protein interaction, whose aim is to achieve a comprehensive and objective global network and present them with a unique set of computational predictions (10). A PPI network analysis was conducted to collect and integrate the different expression of CBXs and potential interactions through STRING.

### Timer

TIMER (https://cistrome.shinyapps.io/timer/) is a user-friendly web interface for 6 major analytic modules to systematic evaluated the infiltration of different immune cells and their clinical impact (11). CBXs was selected to input via “Gene module” and generated scatterplots to visualize the correlation of its expression with immune infiltration level in colon cancer and rectal cancer.

## Results

### Differential Expression of CBXs in CRC Patients

**Figure 1:**
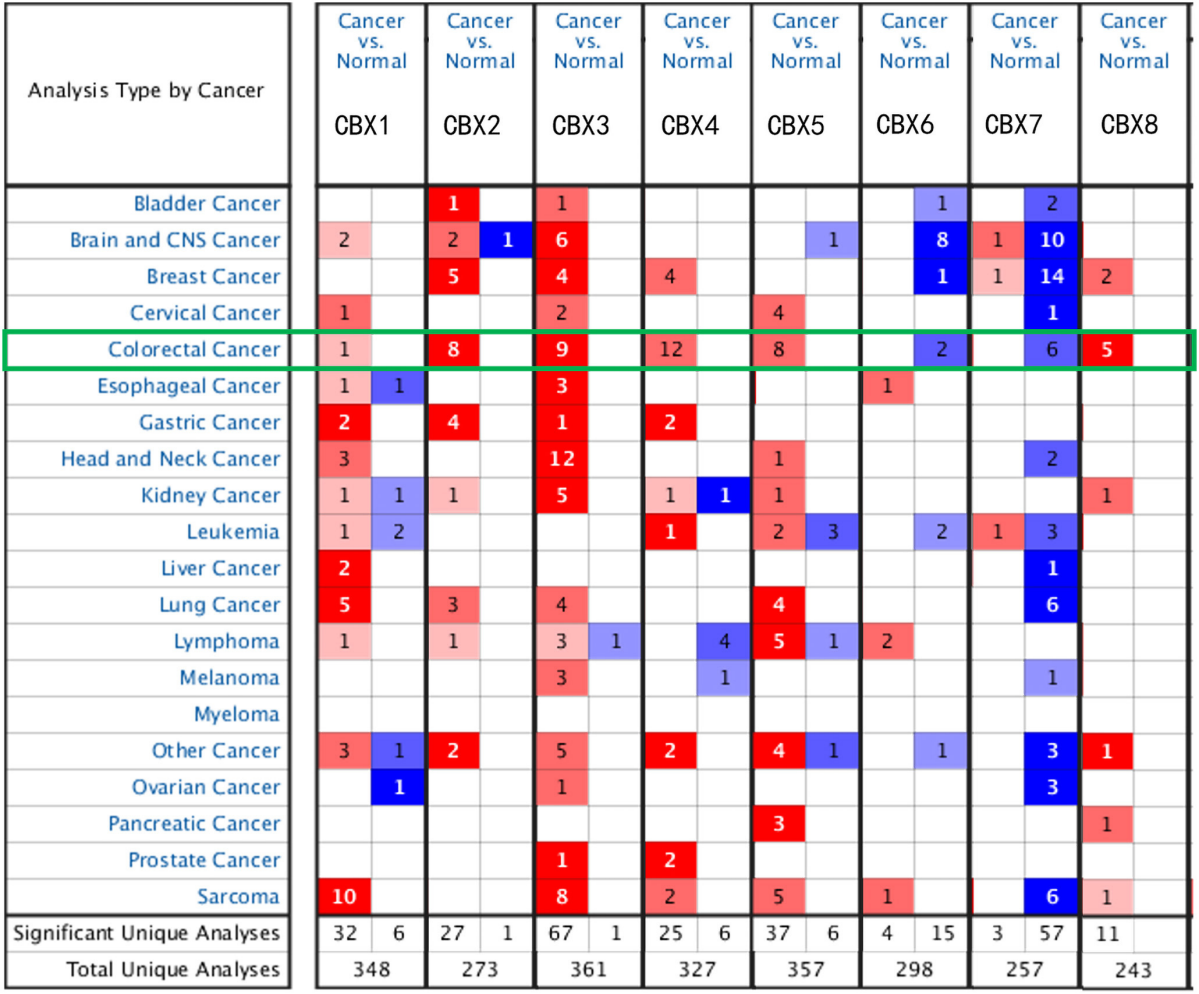
mRNA expression of CBX family members in different cancer types (Oncomine). The graphic demonstrates the numbers of datasets with statistically significant alterations in the mRNA expression of the target gene: upregulated (red) and downregulated (blue). The following criteria were used: *p*-value: 0.01, fold change: 2, gene rank: 10%, data type: mRNA, analysis type: cancer vs. normal tissue. As shown in the green frame, transcriptional levels of CBX1, CBX2, CBX3, CBX4, CBX5, and CBX8 were significantly elevated in CRC tissues, while the transcriptional levels of CBX6 and CBX7 were reduced in CRC vs. normal tissues.

To explore the distinct expression of different CBXs in CRC patients, mRNA expression was analyzed with the Oncomine database (Figure 1). Based on the data obtained from Oncomine, the transcriptional levels of CBX1, CBX2, CBX3, CBX4, CBX5, and CBX8 were significantly elevated in CRC tissues, while the transcriptional levels of CBX6 and CBX7 were reduced in CRC vs. normal tissues. For instance, CBX1 was overexpressed in CRC tissues compared with normal tissues, with a fold change of 2.308 (*p* = 5.78E-04). CBX2 was also significantly upregulated in CRC tissues compared to normal tissues. The results from the TCGA dataset showed a fold change of 4.839 (*p* = 1.00E-14) (Supplementary Table 1).

**Figure 2:**
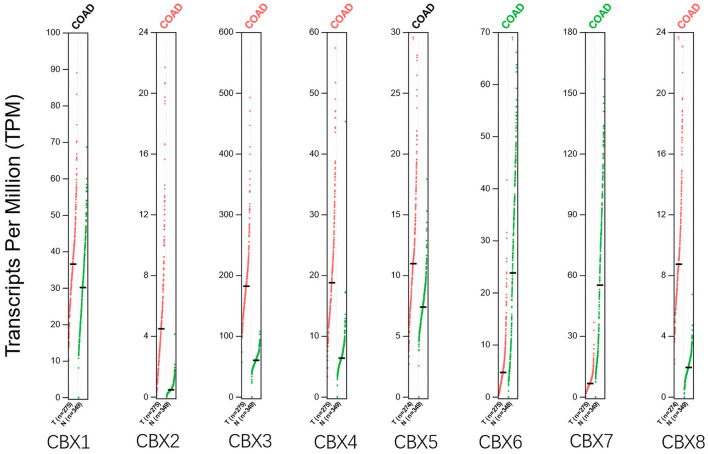
Expression of CBXs in CRC (GEPIA). Scatter diagram demonstrated that the expression levels of CBX2, CBX3, CBX4, and CBX8 were higher in CRC tissues than in normal tissues, and the expression levels of CBX6 and CBX7 were lower in the former than the latter (*p* < 0.05).

Using the GEPIA dataset, we compared the mRNA expression of CBXs between CRC and normal colorectal tissues. The results indicated that the expression levels of CBX2, CBX3, CBX4, and CBX8 were higher in CRC tissues than in normal tissues, and the expression levels of CBX6 and CBX7 were lower in the former than the latter (Figure 2). These results were almost consistent with those from Oncomine.

**Figure 3:**
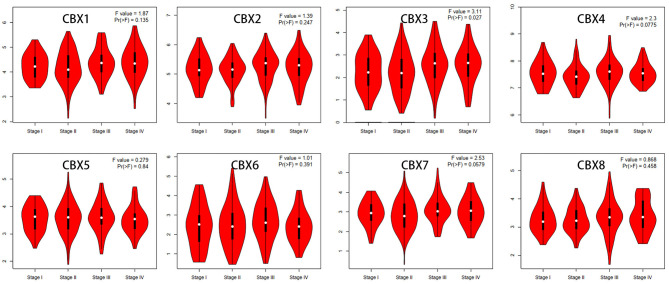
Correlations between CBX expression and tumor stage in colorectal cancer patients (GEPIA). The expression of CBX3 was correlated with the pathological stage of CRC patients (*p* < 0.05).

We then assessed the correlation between the expression of differentially expressed CBXs and the pathological stage of CRC patients. The CBX3 group was highly variable, whereas the CBX1, CBX2, CBX4, CBX5, CBX6, CBX7, and CBX8 groups were not markedly different (Figure 3). These data suggested that CBX3 might play a significant role in the tumorigenesis and progression of CRC.

### Prognostic Value of the mRNA Expression of CBXs in CRC Patients

**Figure 4:**
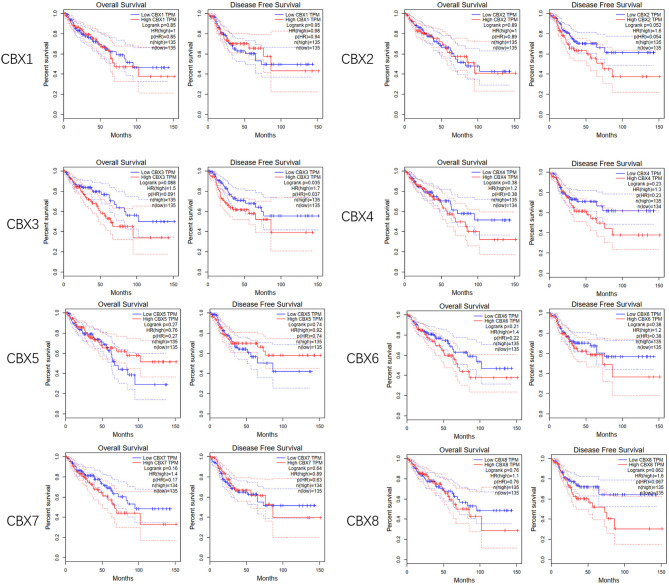
Prognostic value of the mRNA expression of distinct CBX family members in CRC (GEPIA). CRC patients with high transcriptional levels of CBX3 (*p* = 0.035) were significantly associated with short DFS.

To evaluate the value of differentially expressed CBXs in the progression of CRC, correlations between different CBXs and clinical outcomes were analyzed using GEPIA. Disease-free survival (DFS) and overall survival (OS) curves are presented in Figure 4. CRC patients with high transcriptional levels of CBX3 (*p* = 0.035) were significantly associated with short DFS. Except for CBX3, other CBX family members did not seem to have a significant effect on OS or DFS.

**Figure 5:**
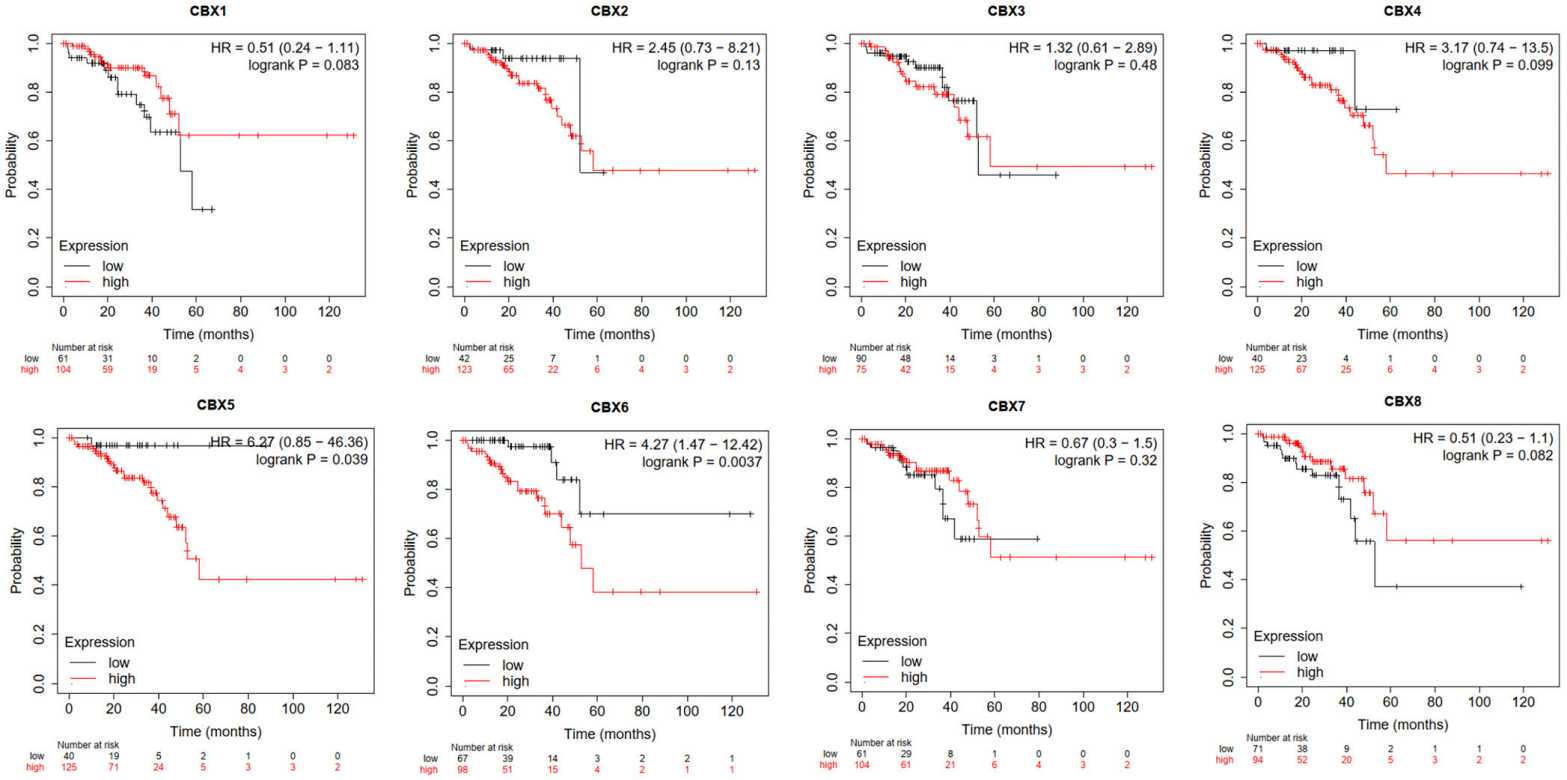
Prognostic value of CBXs in rectal cancer (Kaplan-Meier plotter). High CBX5 and CBX6 mRNA expression was significantly correlated with short OS in rectal cancer patients.

Furthermore, we used Kaplan-Meier plotter to analyze the prognostic values of CBXs in patients with rectal cancer, a subgroup analysis (Figure 5). High CBX5 (HR = 6.27, *p* = 0.039) and CBX6 (HR = 4.27, *p* = 0.0037) mRNA expression was significantly correlated with short OS in rectal cancer patients.

### Genetic Alteration, Expression, and Interaction Analyses of CBXs in CRC Patients

**Figure 6:**
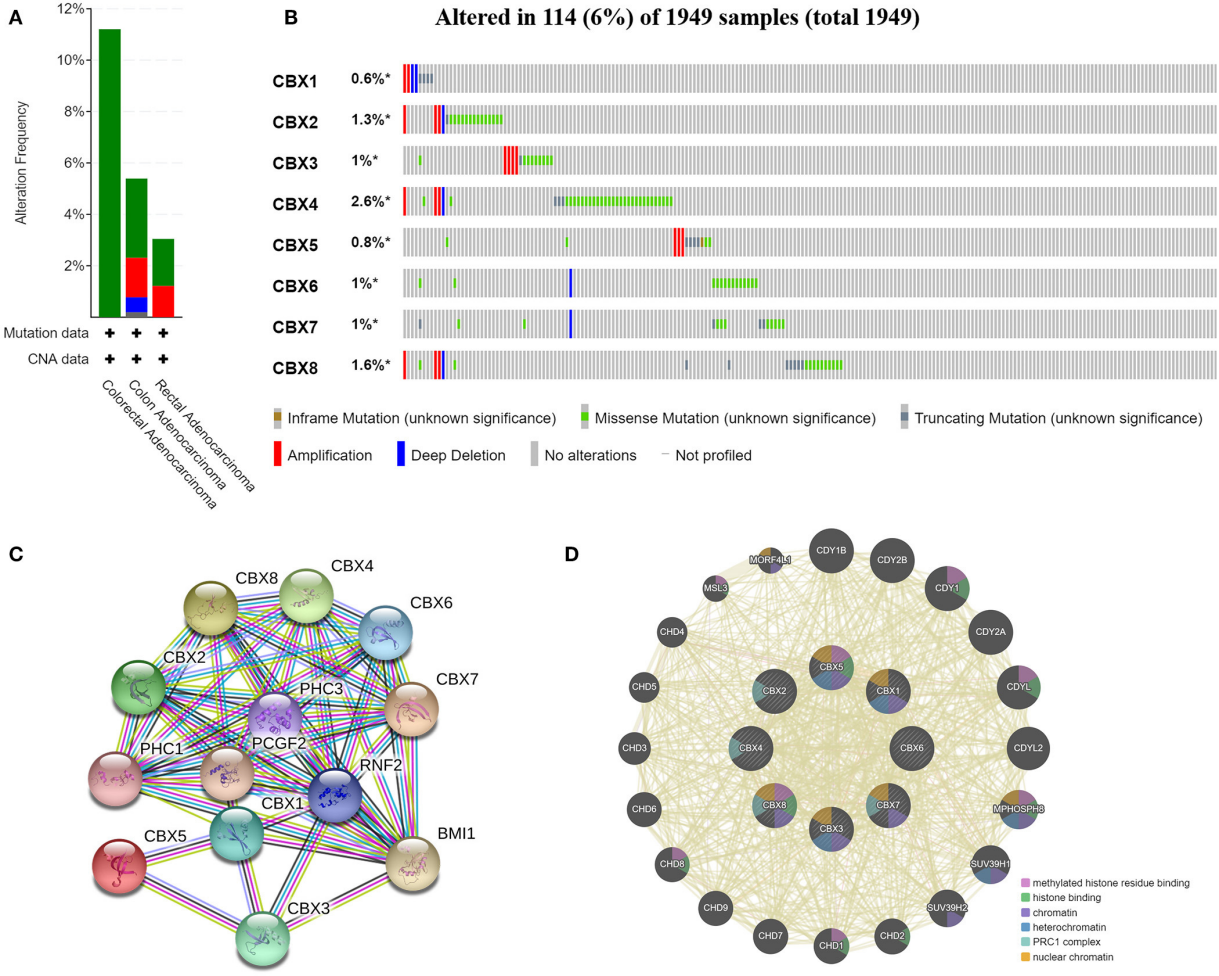
CBX gene mutation and expression analyses in CRC (cBioPortal and STRING). **(A,B)** Summary of alterations in different expressed CBXs in CRC. CBXs were altered in 114 samples of 1,959 patients with CRC, accounting for 16%. **(C,D)** Protein–protein interaction network of different expressed CBXs.

We analyzed the genetic alterations of CBXs in CRC patients by using the cBioPortal online tool. Overall, two or more alterations were detected in different subtypes of CRC, and depletion alterations were more common in rectal cancer samples (Figure 6A). CBXs were altered in 114 samples of 1,959 patients with CRC, accounting for 16% (Figure 6B). In addition, CBX1, CBX2, CBX3, CBX4, CBX5, CBX6, CBX7, and CBX8 were altered in 0.6, 1.3, 1, 2.6, 0.8, 1, 1, and 1.6% of the queried rectal cancer samples, respectively.

Moreover, we conducted a protein-protein interaction PPI network analysis of the differentially expressed CBXs with STRING to explore the potential interactions among them. As expected, several nodes (13) and edges (54) were obtained in the PPI network (Figure 6C). These differentially expressed CBXs were associated with signaling pathways regulating the pluripotency of stem cells. The GeneMANIA results also revealed that the functions of the differentially expressed CBXs and their associated molecules (such as CDY1B, CDY2B, CDY1, CDY2A, CDYL, MPHOSPH8, HUV39H1, CHD2, and CHD1) were primarily related to histone binding, nuclear chromosome, heterochromatin, and the PRC1 complex (Figure 6D).

### Immune Cell Infiltration of CBXs in Patients With Colon Cancer and Rectal Cancer

**Figure 7:**
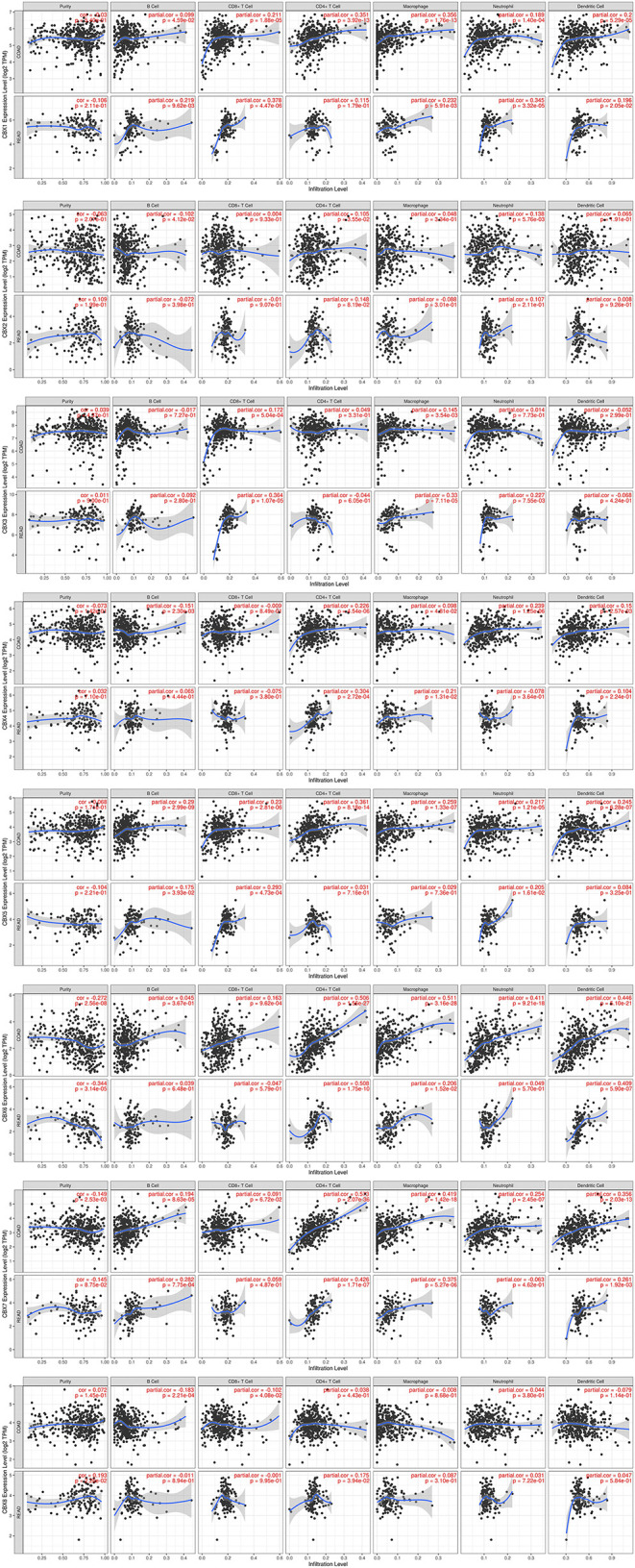
Correlations between differentially expressed CBXs and immune cell infiltration (TIMER). Correlations between the abundance of immune cells and the expression of CBX1-8.

Immune cell level is associated with proliferation and progression of cancer cell. In this study, the TIMER database are used to explore the correlation between CBX members and immune cell infiltration (Figure 7). The expression of CBX1 was in connection with the infiltration of CD4^+^ T cells, neutrophils, B cells, CD8^+^ T cells, macrophages, and dendritic cells in colon and rectal cancers. CBX2 was positively associated with the infiltration of B cells, CD4^+^ T cells, and neutrophils in colon cancer patients, but there was no significant correlation in rectal cancer patients. The expression of CBX3 was positively associated with the infiltration of CD8^+^ T cells and macrophages in the two cancer types. Additionally, the infiltration of B cells, CD4^+^ T cells, macrophages, neutrophils, and dendritic cells was kept in with CBX4 expression in colon cancer patients. With regard to CBX5, all six host immune cells have a positive correlation in colon cancer patients, while the infiltration of B cells, CD8^+^ T cells and neutrophils have a positive correlation with CBX5 in rectal cancer patients. In colon cancer, CBX6 expression was shown to have a positive relation with the infiltration of the all immune cell types except for B cells. In rectal cancer, CBX6 expression was positively correlated with the infiltration of CD4+ T cells, macrophages, and dendritic cells. However, CBX8 expression was is closely tied to only with the infiltration of B cells and CD4^+^ T cells in rectal cancer.

## Discussion

Colorectal cancer is the third most common cancer and remains the leading cause of cancer-related deaths in the world (12). It is now widely accepted that CRC develops from premalignant lesions through a long-term, multistep process. As a heterogeneous disease, well-characterized genetic and epigenetic alterations are accumulated step by step as CRC develops (13). Alterations of target oncogenes, tumor suppressor genes and genes related to DNA repair mechanisms undoubtedly contribute to colorectal carcinogenesis (14). In tumorigenesis of CRC, mutation or aberrant activation of key molecules in the signaling pathway such as Wnt signaling, mitogen-activated protein kinase (MAPK), and phosphatidylinositol 3-kinase (PI3K) signaling, can cause the occurrence of colorectal cancer. Detection of some genes such as c-MYC, KRAS, BRAF, PIK3CA, PTEN can be used as a predictor of colorectal cancer (15). Furthermore, data from the past couple of decades has unequivocally illustrated that epigenetic alterations are important molecular hallmarks, which occur early and manifest frequently. Epigenetic alterations are frequently found in cancers including aberrant DNA methylation, abnormal histone modifications, and altered expression levels of various non-coding RNAs. Therefore, CBX family proteins, which serve as indispensable part of polycomb group complexes, may be involved in epigenetic regulation via modifying the chromatin. Although CBXs have been confirmed to play key roles in breast cancer and hepatic cancer, the distinct roles of CBX family members in CRC remain to be elucidated. In this study, CBXs in CRC were analyzed comprehensively in terms of expression, mutation, prognostic value, and immune cell infiltration.

From the horizontal aspect, CBXs were significantly differentially expressed in CRC. The expression of CBX3 in CRC tissues was higher than that in normal tissues, and its expression was markedly correlated with clinical tumor stage in CRC patients. Moreover, CRC patients with high transcriptional levels of CBX3 were significantly associated with short DFS. These results indicate that CBX3 functions as an oncogene and might take a significant part in the tumorigenesis and progression of CRC. Significant upregulation of CBX3 has been found in a variety of cancers, including lung adenocarcinoma (LUAD), non-small cell lung cancer (nSCLC), and tongue squamous cell carcinoma (TSCC). CBX3 is one of the most frequently overexpressed and amplified histone reader proteins in human LUAD, and high CBX3 mRNA levels are associated with a poor prognosis in LUAD patients. CBX3 promotes the proliferation, colony formation, and migration of LUAD cells (16). CBX3 is overexpressed in human CRC, and it promotes the proliferation of CRC cell lines by directly regulating CDKN1A in a manner associated with the methylation of histone H3K9 on its promoter. Moreover, miR-30a targets CBX3 *in vitro* and *in vivo* to specifically inhibit the growth of CRC in mouse xenograft models, identifying a new miR-30a/CBX3/p21 regulatory axis controlling CRC development (17). As a result, CBX3 participates in the tumorigenesis of CRC and may serve as a potential prognostic biomarker in CRC.

CBX5 also deserves attention in many kinds of malignancies. In breast cancer patients, high CBX5 expression is correlated with decreased survival and the increased occurrence of metastasis over time (18). CBX5 regulates the stem-like properties of lung tumor stem-like cells (TSLCs) and is predictive of lung cancer prognosis, as determined by the identification of target genes based on modeling epistatic signaling mechanics via a predictive and scalable network-based survival model (19). In our study, high CBX5 mRNA expression was significantly correlated with short OS in patients with rectal cancer. These results illustrate that CBX5 plays an oncogenic role in a subtype of CRC. Similarly, CBX6 showed the same characteristics in patients with rectal cancer, although its expression was increased in tumor tissues compared to normal tissues. Further verification is needed to determine whether CBX6, like other CBX family members, plays an oncogenic role in CRC.

Further genetic analysis indicated frequent genetic alterations in the CBXs that are differentially expressed in CRC. In this study, the functions of these genes are found to be primarily related to the SUMOylation (small ubiquitin-related modifier) of DNA methylation proteins, the SUMOylation of chromatin organization proteins, the SUMOylation of RNA binding proteins (Reactome) and signaling pathways that regulate the pluripotency of stem cells [Kyoto Encyclopedia of Genes and Genomes (KEGG)].

Tumor microenvironment (TME) is becoming an increasingly popular topic and could affect tumor progression and recurrence. Immune cells in TME have been shown to harbor either tumor-promoting or tumor-suppressing activities. They are considered as a significant determining factor of both clinical outcome and the response to immunotherapy (20, 21). Our study showed that the expression of CBXs might be significantly correlated with and the infiltration of six immune cell types, suggesting that CBXs may also reflect the immune status besides the disease prognosis. This study might provide detailed immunization information to assist in the design of new immunotherapies.

There were some limitations to our study. All the data analyzed in our study were retrieved from online databases, and further studies consisting of cell experiments and clinical studies are required to validate our findings and to further explore the potential mechanisms, molecules interactions, and clinical applications of distinct CBXs in CRC.

## Conclusion

In conclusion, we systematically analyzed the differential expression and prognostic value of CBX family members in CRC. The expression levels of CBX1, CBX2, CBX3, CBX4, CBX5, and CBX8 were significantly elevated in CRC tissues, while the expression levels of CBX6 and CBX7 were reduced. CBX3 was found to be significantly associated with the clinical cancer stage and short DFS in CRC patients. High mRNA expression of CBX5/6 was associated with short OS in rectal cancer patients. These results indicate that CBX3/5/6 could be potential prognostic biomarkers for the survival of CRC patients. Moreover, the functions of the differentially expressed CBXs are mainly involved in the SUMOylation of DNA methylation proteins and chromatin organization and may regulate the pluripotency of stem cells. The expression of CBXs were found to be significantly correlated with the infiltration of diverse immune cells, including six types of CD4+ T cells, macrophages, neutrophils, B cells, CD8+ T cells, and dendritic cells in CRC cancers. Our study may provide novel ideas and comprehensive analysis to select prognostic biomarkers among CBX family members in colorectal cancer.

## Data Availability Statement

All datasets generated for this study are included in the article/Supplementary Material.

## Author Contributions

QL and YP study design and bioinformatics analysis. ZC and SZ critical revision of the manuscript. All authors contributed to manuscript revision, read, and approved the submitted version.

## Conflict of Interest

The authors declare that the research was conducted in the absence of any commercial or financial relationships that could be construed as a potential conflict of interest.

## Abbreviations

CRC: colorectal cancer
PcG: polycomb group
CBXs: chromobox family members
TCGA: The Cancer Genome Atlas
PPI: protein-protein interaction
OS: overall survival
DFS: disease-free survival
TSLCs: tumor stem-like cells
LUAD: lung adenocarcinoma
nSCLC: non-small cell lung cancer
TSCC: tongue squamous cell carcinoma
COAD: colorectal adenocarcinoma.
