# Supplementary material for: Comprehensive Analysis of Prognostic Value and Immune Infiltration of Chromobox Family Members in Colorectal Cancer
Source: Front Oncol. 2020 Sep 4;10:582667. doi: 10.3389/fonc.2020.582667 (PMC7498700; doi:10.3389/fonc.2020.582667)
Supplement: Supplementary file 1 [file Table_1.DOCX]

**Supplemental Material**

| **Supplemental table 1**: The mRNA levels of CBXs in different types of CRC tissues and normal tissues at transcriptome level. | | | | | |
| --- | --- | --- | --- | --- | --- |
| **Name** | **Types of CRC VS. Nomal tissues** | **Fold Change** | **P Value** | **t-test** | **Reference or Source** |
| **CBX1** | Rectal Mucinous Adenocarcinoma | 2.308 | 5.78E-04 | 4.283 | TCGA |
| **CBX2** | Rectal Mucinous Adenocarcinoma | 4.839 | 1.00E-14 | 15.457 | TCGA |
|  | Colon Mucinous Adenocarcinoma | 3.301 | 7.37E-11 | 8.527 | TCGA |
|  | Cecum Adenocarcinoma | 4.254 | 2.53E-10 | 8.525 | TCGA |
|  | Rectal Adenocarcinoma | 3.581 | 1.88E-15 | 10.574 | TCGA |
|  | Colon Adenocarcinoma | 3.083 | 5.78E-14 | 10.13 | TCGA |
|  | Colon Carcinoma Epithelia | 2.872 | 6.17E-09 | 15.795 | Skrzypczak M(1) |
|  | Colorectal Carcinoma | 2.236 | 3.52E-10 | 7.724 | Skrzypczak M(1) |
|  | Colorectal Carcinoma | 14.991 | 7.99E-10 | 12.207 | Hong Y(2) |
| **CBX3** | Colon Adenocarcinoma | 2.232 | 9.16E-07 | 5.415 | Alon U(3) |
|  | Rectal Adenoma | 2.282 | 2.62E-11 | 14.401 | Sabates-Bellver J(4) |
|  | Colon Adenoma | 2.01 | 1.47E-10 | 8.308 | Sabates-Bellver J(4) |
|  | Colon Adenocarcinoma | 2.293 | 1.14E-06 | 5.869 | Notterman DA(5) |
|  | Rectal Adenocarcinoma | 2.123 | 6.91E-05 | 5.816 | Kaiser S(6) |
|  | Colon Adenocarcinoma | 2.375 | 3.90E-08 | 8.553 | Kaiser S(6) |
|  | Cecum Adenocarcinoma | 2.139 | 3.22E-06 | 6.146 | Kaiser S(6) |
|  | Colorectal Carcinoma | 2.587 | 1.79E-10 | 12.915 | Hong Y(2) |
|  | Rectosigmoid Adenocarcinoma | 2.645 | 3.50E-04 | 11.521 | TCGA |
| **CBX4** | Colon Carcinoma Epithelia | 3.584 | 2.60E-09 | 13.464 | Skrzypczak M(1) |
|  | Colon Carcinoma | 4.064 | 1.44E-08 | 11.722 | Skrzypczak M(1) |
|  | Colon Adenoma | 2.327 | 1.92E-05 | 6.283 | Skrzypczak M(1) |
|  | Rectosigmoid Adenocarcinoma | 3.075 | 4.29E-06 | 7.083 | Kaiser S(6) |
|  | Rectal Mucinous Adenocarcinoma | 2.635 | 8.68E-05 | 9.679 | Kaiser S(6) |
|  | Colon Mucinous Adenocarcinoma | 2.428 | 1.07E-05 | 6.666 | Kaiser S(6) |
|  | Cecum Adenocarcinoma | 2.336 | 2.21E-05 | 7.237 | Kaiser S(6) |
|  | Colon Adenocarcinoma | 2.095 | 1.56E-16 | 12.188 | TCGA |
|  | Cecum Adenocarcinoma | 2.067 | 5.12E-10 | 7.899 | TCGA |
|  | Colon Mucinous Adenocarcinoma | 2.122 | 7.60E-09 | 7.22 | TCGA |
|  | Rectal Adenocarcinoma | 2.046 | 1.70E-12 | 8.544 | TCGA |
|  | Colorectal Carcinoma | 3.645 | 7.13E-09 | 10.147 | Hong Y(2) |
| **CBX5** | Rectal Mucinous Adenocarcinoma | 4.25 | 2.80E-05 | 9.485 | Kaiser S(6) |
|  | Rectal Adenocarcinoma | 3.338 | 3.08E-05 | 6.502 | Kaiser S(6) |
|  | Rectosigmoid Adenocarcinoma | 3.263 | 3.03E-05 | 6.013 | Kaiser S(6) |
|  | Cecum Adenocarcinoma | 3.421 | 9.36E-06 | 6.042 | Kaiser S(6) |
|  | Colon Adenoma Epithelia | 2.482 | 7.49E-07 | 9.881 | Skrzypczak M(1) |
|  | Colon Adenoma | 3.281 | 3.96E-06 | 9.211 | Skrzypczak M(1) |
|  | Colon Carcinoma | 2.169 | 1.04E-06 | 8.057 | Skrzypczak M(1) |
|  | Rectal Adenoma | 3.678 | 3.92E-05 | 5.132 | Sabates-Bellver J(4) |
| **CBX8** | Cecum Adenocarcinoma | 2.322 | 5.48E-16 | 12.487 | TCGA |
|  | Colon Adenocarcinoma | 2.652 | 4.08E-21 | 17.903 | TCGA |
|  | Rectal Adenocarcinoma | 2.326 | 9.09E-20 | 13.732 | TCGA |
|  | Colon Mucinous Adenocarcinoma | 2.287 | 1.23E-11 | 9.569 | TCGA |
|  | Rectal Adenocarcinoma | 2.444 | 2.58E-34 | 19.395 | Gaedcke J(7) |

1. Skrzypczak M, Goryca K, Rubel T, Paziewska A, Mikula M, Jarosz D, et al. Modeling oncogenic signaling in colon tumors by multidirectional analyses of microarray data directed for maximization of analytical reliability. *PLoS One* (2010) 5(10). Epub 2010/10/20. doi: 10.1371/journal.pone.0013091. PubMed PMID: 20957034; PubMed Central PMCID: PMCPMC2948500.

2. Hong Y, Downey T, Eu KW, Koh PK, Cheah PY. A 'metastasis-prone' signature for early-stage mismatch-repair proficient sporadic colorectal cancer patients and its implications for possible therapeutics. *Clinical & experimental metastasis* (2010) 27(2):83-90. Epub 2010/02/10. doi: 10.1007/s10585-010-9305-4. PubMed PMID: 20143136.

3. Alon U, Barkai N, Notterman DA, Gish K, Ybarra S, Mack D, et al. Broad patterns of gene expression revealed by clustering analysis of tumor and normal colon tissues probed by oligonucleotide arrays. *Proc Natl Acad Sci U S A* (1999) 96(12):6745-50. Epub 1999/06/09. doi: 10.1073/pnas.96.12.6745. PubMed PMID: 10359783; PubMed Central PMCID: PMCPMC21986.

4. Sabates-Bellver J, Van der Flier LG, de Palo M, Cattaneo E, Maake C, Rehrauer H, et al. Transcriptome profile of human colorectal adenomas. *Molecular cancer research : MCR* (2007) 5(12):1263-75. Epub 2008/01/04. doi: 10.1158/1541-7786.Mcr-07-0267. PubMed PMID: 18171984.

5. Notterman DA, Alon U, Sierk AJ, Levine AJ. Transcriptional gene expression profiles of colorectal adenoma, adenocarcinoma, and normal tissue examined by oligonucleotide arrays. *Cancer Res* (2001) 61(7):3124-30. Epub 2001/04/18. PubMed PMID: 11306497.

6. Kaiser S, Park YK, Franklin JL, Halberg RB, Yu M, Jessen WJ, et al. Transcriptional recapitulation and subversion of embryonic colon development by mouse colon tumor models and human colon cancer. *Genome biology* (2007) 8(7):R131. Epub 2007/07/07. doi: 10.1186/gb-2007-8-7-r131. PubMed PMID: 17615082; PubMed Central PMCID: PMCPMC2323222.

7. Gaedcke J, Grade M, Jung K, Camps J, Jo P, Emons G, et al. Mutated KRAS results in overexpression of DUSP4, a MAP-kinase phosphatase, and SMYD3, a histone methyltransferase, in rectal carcinomas. *Genes, chromosomes & cancer* (2010) 49(11):1024-34. Epub 2010/08/21. doi: 10.1002/gcc.20811. PubMed PMID: 20725992; PubMed Central PMCID: PMCPMC3535184.
